# ‘It is a hard decision’: a qualitative study of perinatal intimate partner violence disclosure

**DOI:** 10.1186/s12978-022-01514-7

**Published:** 2022-11-14

**Authors:** Malikeh Amel Barez, Khadijeh Mirzaii Najmabadi, Robab Latifnejad Roudsari, Mojtaba Mousavi Bazaz, Raheleh Babazadeh

**Affiliations:** 1 Nursing and Midwifery Care Research Center, Mashhad University of Medical Sciences, Mashhad, Iran; 2 Department of Midwifery, Faculty of Nursing and Midwifery, Mashhad Medical Sciences, Islamic Azad University, Mashhad, Iran; 3 Department of Midwifery, School of Nursing and Midwifery, Mashhad University of Medical Sciences, Mashhad, Iran; 4 Department of Community Medicine, Faculty of Medicine, Mashhad University of Medical Sciences, Mashhad, Iran

**Keywords:** Intimate partner violence, Disclosure, Barriers, Facilitators, Perinatal, Qualitative study

## Abstract

**Background:**

Perinatal intimate partner violence is a hidden under reported and difficult to identify problem which has negative effects on mother and child. The present study aimed to explore barriers and facilitators of perinatal intimate partner violence disclosure.

**Methods:**

This qualitative study was carried out from October 2019 to January 2021 in Mashhad, Iran. Participants included 23 abused women (11 pregnant and 12 after birth) which were selected via purposive sampling. Semi-structured in-depth interviews and focus group discussion were conducted until the data saturation was achieved. The data analysis was performed based on conventional content analysis adopted by Graneheim & Lundman.

**Results:**

The main themes “barriers to disclosure” and “facilitators of disclosure” were emerged as the result of data analysis. Barriers to disclosure included negative disclosure consequences and protection of family privacy. Facilitators of disclosure included maternal self-efficacy, threats to security, and formal and informal supportive networks.

**Conclusions:**

Most abused women did not disclose violence despite routine screening for perinatal intimate partner violence in antenatal care. Recognizing the barriers to and facilitators of violence disclosure play an important role in eliminating barriers, strengthening facilitators, providing effective supportive services for abused women, and reducing perinatal violence. Focus on the barriers to and the facilitators of disclosure will be useful to policymakers, health program planners, and health care providers to identify and manage intimate partner violence, appropriately.

**Supplementary Information:**

The online version contains supplementary material available at 10.1186/s12978-022-01514-7.

## Plain language summary

Disclosure of perinatal intimate partner violence is a difficult decision. Several barriers prevent abused pregnant women from disclosing violence. The present study explained barriers and facilitators of perinatal intimate partner violence disclosure. 23 women (11 pregnant and 12 after birth) who experienced perinatal intimate partner violence were interviewed in Mashhad, Iran. The results showed the barriers to disclosure include negative disclosure consequences and protection of family privacy and the facilitators of disclosure include maternal self-efficacy, threats to security, and formal and informal supportive networks. In conclusion eliminating barriers and strengthen facilitators play an important role in providing effective supportive services for abused women and reducing perinatal violence. The result will be useful to policymakers, health program planners, and health care providers for appropriate management of perinatal intimate partner violence.

**Supplementary Information:**

The online version contains supplementary material available at 10.1186/s12978-022-01514-7.

## Background

Perinatal intimate partner violence is a common and serious global public health problem and human rights violation that adversely affects the health of women and their offspring [1, 2]. The World Health Organization defines intimate partner violence (IPV) as physical, emotional, sexual abusive acts, and controlling behaviors performed by a present or previous intimate partner [3]. Perinatal intimate partner violence includes violence occurring 12 months before pregnancy, during pregnancy, and up to one year after delivery by an intimate partner [4–7]. Universal studies have found differences in the prevalence of IPV during pregnancy, ranging from 1 to 20% [8, 9], depending on the definition, cultural differences, context, and methodology used in exploring violence [10–12]. The overall prevalence of intimate partner violence in Iranian pregnant women was 52% with the prevalence rates of 19% physical, 45% psychological, and 31% sexual violence which is dependent on the level of couples education, their job, and the sociocultural level [13]. IPV may initiate or deteriorate during pregnancy and the postpartum period [14]. A global review study indicated that 13–71% of women reported an increase in the frequency or severity of violence during pregnancy [15].

Pregnancy alone imposes significant psychological and physical stress on a woman, and when accompanied by other stress factors such as violence, they can adversely affect the health of mother and child which can increase maternal and neonatal morbidity and mortality [16]. Adverse consequences included inadequate antenatal weight gain, miscarriage, preterm labor, vaginal bleeding, preeclampsia, dystocia, preterm and low birth weight infants, and postpartum depression [17, 18].

It is important to understand when women disclose IPV, particularly given that at least 20 percent of those who experience violence tell no one else about it [13]. Early identification of women experiencing IPV is the first step for screening and intervening to maintain their safety and well-being in many health systems [19, 20]. Prenatal care is an opportunity for healthcare providers to identify abused pregnant women and enable appropriate counseling and intervention programs to protect the health of mothers and infants [21]. Based on the literature, some facilitators of disclosure include clinician’s awareness of IPV, privacy, and non-judgmental attitudes [22]. Furthermore, positive relationships with the healthcare professionals, direct questioning, and making abused women ensure their disclosure is confidential could help disclosure [23]. Barriers to disclosure included negative attitudes of health care professionals, abused women's concerns about the consequences of disclosing [23], as well as their fear, shame and self-blame, and loss of financial security [24].

Iranian society is patriarchal and emphasizes men’s domination over women in the family. Women must obey their husbands, tolerate violence, and maintain the family [25]. Most of the women are financially dependent on their husbands [26]. Domestic roles define women as wives or mothers, and both of these roles expect them to put their family needs ahead of their own to maintain the family from the danger of collapsing [27]. Therefore, it is not a surprise that the social-cultural environment of Iran would lead abused women not seeking help and not leave their marriages. Iranian abused women refused to disclose spouse violence for the fear of exposing disclosure consequences, such as divorce, losing custody of the children, and the difficulties of living alone [28]. Regarding the influence of contextual factors in intimate partner violence, it is essential to explore the subject in the context of Iranian society from different socio-cultural perspectives. Therefore, this qualitative study aimed to explore barriers and facilitators of perinatal intimate partner violence disclosure.

### Theoretical framework

Several theoretical perspectives attempt to explain intimate partner violence in women’s lives. However, the specificities of perinatal intimate partner violence remain poorly understood. Feminist theory and stress related to pregnancy seem to be suitable theoretical frameworks for a better understanding of perinatal intimate partner violence [15, 29]. The feminist theory considers power and control to be tools to maintain male domination over women in a patriarchal social system [30]. Feminist theory is appropriate to explain some of patriarchy consequences. Patriarchy refers to “power of fathers” in which men occupy the largest proportion of power and control within the family [31]. Evidence indicates that intimate partner violence is a frequent occurrence in societies and families where high levels of gender inequality exist, women have less power and male partners have greater authority and control [32]. The patriarchal social system is perceived by many feminist perspectives as justifying and condoning physical violence against women. Victims of intimate partner violence during pregnancy experience various degrees of “coercive control” by their intimate partners. Different family structures may affect one’s experiences of strain. Therefore, the strain is proposed to occur among intimate partners during pregnancy, leading to a higher risk of IPV [33]. Pregnancy related factors such as unplanned and unwanted pregnancy, young age at the time of pregnancy, economic difficulties, and changing the social role of men and women when they become parents increase the stress experienced by the couple which in turn may increase the risk for intimate partner violence during pregnancy [15]. These theoretical frameworks provided a theoretical foundation to understand the barriers and facilitators of perinatal intimate partner violence disclosure among Iranian women.

## Methods

### Study design

The conventional content analysis approach was used to design this qualitative study to get a deeper insight into the barriers and facilitators of perinatal intimate partner violence disclosure [34]. Qualitative content analysis is a proper method to study cultural related contextual issue [35] in health science studies [36].

### Setting

This study was conducted from October 2019 to January 2021 in Mashhad, the second-most-populous city in northeast of Iran and the capital of Khorasan-e Razavi Province. At first, different departments of 17 Shahrivar Hospital, such as triage, delivery, obstetrics, gynecology, and prenatal clinic, were used to select the participants. The reasons for the selection of this hospital was the high coverage of pregnant women, effective management of these departments, and the long-term presence of the first researcher at this hospital. In addition to 17 Shahrivar Hospital, purposive sampling led to select Health Centers, Forensic Medicine Center, Provincial Welfare Center, Social Emergency, Consultant Voice Center, Midwifery Counseling Center, Prenatal Clinic, Comprehensive Health Center, Midwifery Department, and Teaching Hospitals as study settings.

### Data collection

Purposive sampling adopting a maximum variation strategy based on age, education, occupation, gestational age, wanted or unwanted pregnancy, and violence screening tool (HITS: Hurt, Insult, Threaten and Scream) was used to select the participants. HITS scale is a short screening tool for the domestic violence and intimate partner violence which contains four questions about violence. Score values could range from 4 to 20 (score more than 10 indicates the existence of violence) [37, 38]. Participants included 23 abused women (11 pregnant and 12 after birth) who experienced perinatal intimate partner violence. Eligibility criteria were pregnant women or those who have given birth with positive HITS screening score, agreement for the participation in the study, ability to communicate in Persian and express feeling, and ability to share the perinatal intimate partner violence-related experiences. The exclusion criteria was physical and mental illnesses that prevent mothers preparing to participate in research.

### Interview procedures

The data were collected via 11 semi-structured face to face in-depth individual interviews. Similar to previous studies [39–41], in the present study with the sensitive topic and hard to reach population, one online focus group discussion with attendance of 10 abused mothers of higher socio-economic level who did not accept to be individually interviewed was conducted via the Telegram. The group was led by the first author and lasted approximately 150 min. The focus group leader was previously qualified on how to manage focus group by the principal investigator of the study. The focus group included questions regarding the overall experiences of women who have been exposed to perinatal intimate partner violence. The first author stated the questions to the group of abused mothers in written via this web based platform and encouraged group members to discuss, respond and interact by typing words.

The individual interviews were performed at a convenient time and place for the participants and were audio-recorded. Notes were taken by the interviewer (the first author). The interviews continued until the data saturation. However, two further interviews were conducted to ensure data saturation, which showed no new data. An interview guide with open-ended and possible follow-up questions were designed to explore the experiences of each participant as follows “Please describe your experience of perinatal intimate partner violence?” Other questions followed the main question were “Under which circumstances do you disclose intimate partner violence?”, and “What factors prevent you from disclosing intimate partner violence?” The mean length of interviews duration was 68 min (range: 30–120 min) (Additional file 1).

### Data analysis

The conventional content analysis approach was used to explain the research question. The data were simultaneously analyzed with data collection, using Graneheim and Lundman method which allows the researchers to examine individual experiences and shows conflicting opinions and unsolved issues regarding the meaning and use of concepts, procedures and interpretation [36, 42, 43] by MAXQDA software (version 10, VERBI Software, Berlin, Germany). After each interview, the first author listened to the interviews several times to obtain a general perspective of their content, and then transcribed the interviews verbatim and read it several times to gain an overall understanding of their content. The text of each interview was divided into meaning units as words, phrases, sentences, and paragraphs. The meaning units were condensed and abstracted, and given a descriptive code. Based on the similarities and differences, codes were classified into subcategories and categories and finally determined the themes.

### Ethical considerations

The research was approved by Local Research Ethics Committee of Mashhad University of Medical Sciences (Code of Ethics: IR.MUMS.NURSE.REC.1398.026). All experimental protocols for involving humans were based on the guidelines of the Declaration of Helsinki in the manuscript [44]. The participants were fully informed about the purpose and nature of the study as well as their voluntary participation. They were reassured that their right to withdraw from the study without any prejudice, also the privacy and the confidentiality of all their data would be maintained. Prior to the start of the interview, conscious written informed consent was obtained from all the participants. Informed consent was obtained from the legal guardian of Illiterate. If any of the questions caused distress for the participants, the interview was stopped, and after a while, and by the participant’s permission, it was continued. After the completion of the interview, the researcher was assured that the participants were not psychologically distressed in terms of the interview and that there was no need for immediate emotional support. At the end of interviews, necessary information about the existing services for abused women was given to the participants, and they were referred to receive services, if necessary. Each participant was given a hypothetical code and name to keep their information confidential.

### Trustworthiness

Lincoln and Guba criteria for credibility, confirmability, dependability, and transferability were applied to verify the trustworthiness of data [45]. To prove the credibility of results, the extracted codes and categories from the data were reviewed and approved by the participants (member check) and three expert co-authors. To increase credibility, we considered maximum variation in sampling. To ensure confirmability, three supervisors reviewed the findings, interpretations and conclusions of the study, making it possible to conduct an audit trial. For dependability, two independent researchers who were experienced in qualitative research checked and approved the research process and the data analysis. For transferability, a distinct and clear description of culture, context, selection of the participant, characteristics of the participants, data collection and data analysis, as well as vigorous presentation of the results and appropriate quotations was provided.

## Results

The participants included 23 abused women who were victims of perinatal intimate partner violence. 19 participants had experienced intimate violence prior to pregnancy. Six abused women had disclosed perinatal intimate partner violence. The abused women’s age ranged from 19 to 41 years. Education levels ranged from illiterate to doctor of philosophy degrees. HITS scores ranged from 11 to 20. The profile of participants is shown in Tables 1 and 2.

**Table 1:** The profile of women participated in the study

Participant	Age of mother/ age of husband	Education of mother /husband	Mother’s job	Husband’s job	Gestational age	Planned/unplanned pregnancy	Self-reported Economic status	Disclosed violence	Violence type
1	37/ 53	8 years/8 years	House wife	Retired	37w	Unplanned	Fairly appropriate	No	Physical Sexual Emotional controlling behavior
2	22/ 29	7 years/ diploma	House wife	Factory worker	39w	Planned	Good	No	Emotional controlling behavior
3	29/ 30	7 years/ diploma	House wife	Unemployed	40w	Planned	Poor	No	Emotional controlling behavior
4	19/ 30	Diploma/Associate Degree	House wife	Factory worker	35w	Planned	Good	Yes	Physical Sexual Emotional
5	25/ 25	Illiterate/ 6 years	House wife	Factory worker	10 h after birth	Unplanned	Fairly appropriate	No	Physical Emotional
6	41/ 47	Diploma/6 years	House wife	Sales manager	45 days after birth	Unplanned	Good	Yes	Physical Sexual Emotional
7	36/ 31	Diploma/6 years	House wife	Driver	17w	Unplanned	Poor	Yes	Physical Emotional controlling behavior
8	24/ 28	Diploma/ diploma	House wife	Factory worker	20w	Unplanned	Good	Yes	Physical Sexual Emotional controlling behavior
9	28/ 26	Diploma/ 6 years	Employed	Private business	8w	unplanned	Good	Yes	Physical Sexual Emotional controlling behavior
10	36/ 35	Doctor of Philosophy / master degree	Factory manager	Factory manager	1 year after birth	Planned	Good	No	Emotional controlling behaviors
11	36/ 40	Master degree/ diploma	Engineer	Self employed	1 year after birth	Planned	Good	No	Emotional controlling behaviors
12	26/ 31	Master degree/ doctorate	teacher	Doctor	39w	Planned	Good	No	Emotional
13	36/ 40	Bachler degree	Employer	Self employed	1 year after birth	Unplanned	Good	No	Emotional Sexual

**Table 2:** The profile of women participated in focus group discussion

Participant	Age of mother/age of husband	Education of mother /husband	Mother’s job	Husband's job	Gestational age	Planned/unplanned pregnancy	Self-reported economic status	Disclosed violence	Violence type
1	27/ 32	Master’s degree/ Bachelor’s degree	Teacher	Engineer	6 months after delivery	Planned	Good	No	Emotional controlling behavior
2	22/ 25	10 years/ diploma	House wife	Private business	34w	Planned	Fairly appropriate	Yes	Physical Emotional Controlling behavior
3	35/ 38	Diploma/Bachelor’s degree	Hair dresser	Private business	38w	Planned	Good	No	Emotional Sexual
4	29/ 30	Bachelor’s degree/ Bachelor’s degree	House wife	Employer	8 months after birth	Planned	Good	No	Physical Emotional controlling behavior
5	30/ 35	Master’s degree/ diploma	Employer	Private business	1 year after birth	Planned	Good	No	Emotional Sexual
6	32/ 38	Doctor of Philosophy/ Master’s degree	Engineer	Engineer	10 months after birth	Planned	Good	No	Emotional
7	28/ 32	Bachelor’s degree/ Master’s degree	Teacher	Teacher	4 months after birth	Planned	Good	No	Emotional Controlling behavior
8	32/ 40	Bachelor’s degree/ Bachelor’s degree	House wife	Employer	6 months after birth	Planned	Good	No	Emotional controlling behavior
9	36/ 42	Master’s degree/ medicine doctorate	Teacher	Doctor	37 weeks	Planned	Fairly appropriate	No	Emotional Sexual
10	38/ 43	Bachelor’s degree/diploma	Employer	Private business	1 year after birth	Planned	Good	No	Emotional

In this study, 958 codes, ten subcategories, five categories, and two main themes emerged from the data analysis. The data analysis procedures identified “barriers to disclosure” and “facilitators of disclosure” as the overarching themes. Barriers to disclosure was comprised of two categories, including “negative disclosure consequences” and “protection of family privacy”. Facilitators of disclosure was comprised of three categories, including “maternal self-efficacy”, “threats to security” and “formal and informal supportive networks”. A more precise presentation of the results is given in Additional file 2: Table S1.

## Main themes

The main themes that emerged from the data analysis were “barriers to disclosure” and “facilitators of disclosure”. Women’s experiences showed that they faced some barriers to disclose perinatal intimate partner violence. Similarly, several factors facilitated violence disclosure.

### Theme 1: barriers to disclosure

Most abused women concealed perinatal intimate partner violence, and only a few women disclosed this situation. Barriers to disclosure included negative disclosure consequences.

### Category 1: negative disclosure consequences

Women usually encountered with several negative consequences when they disclosed perinatal intimate partner violence. These negative consequences prevented them from disclosing violence and included facing with multiple fears and concerns about social judgments.

#### Facing with multiple fears

Abused women faced with several fears that prevented them from disclosing perinatal violence. Fear of not believed, difficult economic conditions, husband’s reaction to disclosure, intensifying violence, being left by her husband, losing child, losing family support and negative reaction of friends, family, or the health care professionals were the barriers of disclosing perinatal intimate partner violence. One of the participants explained about the fear of intensifying violence and losing child as a disclosure barriers:

*“I did not tell violence to anyone because I was afraid of my husband’s announcement and increasing his aggressive behavior. If my husband finds out that I told someone about his violence, he will give the child from me and kick me out of the house.” (FGD- participant 7)*

#### Concern about social judgments

Most of the women faced a set of social judgments and their consequences after informing others about their spouse’s violence. These social judgments included Loss of social reputation, social isolation, the stigma of violence, divorce and remarriage, and the shame and blame of violence that have led to the concealment of violence. This quote reflects the stigma of divorce and remarriage:

*“…I don’t want to be a widow. It is not good to go back to my father’s house. A second marriage with a child will be difficult for me, so I must concealed my husband’s violence.” (Participant 5)*

Some abused women concealed their husbands’ violence to maintain their social credibility and reputation against their family, relatives, and friends. Concern about the children’s reputation and commitment to their husbands were other effective factors in concealing intimate partner violence. One educated mother explained about maintaining her social reputation and credibility by concealment of her spouse’s violence:

*“…I hide violence from others for the sake of my reputation, to pretend I have nothing less than others.” (FGD- participant 8)*

### Category 2: protection of family privacy

The result of the present study showed that protection of family privacy by maintaining maternal commitment and protection the unborn baby made the abused women to conceal perinatal intimate partner violence.

#### Protection of the unborn baby

Protection of the unborn baby such as prevention future stigmatization of the child and protection father’s presence for the children was the barrier of disclosing perinatal intimate partner violence. One mother explained:

*“I don’t tell my husband’s violence to anyone because I want to protect my baby, I want to prevent the stigmatization of my child in the future.” (Participant 7)*

#### Maternal commitments

Maternal commitments, such as the feeling of being a mother, maintaining marital life and giving priority to children’s comfort, made the mother hide her husband’s violence and stay in an abusive relationship. The following statement indicated:

*“When you become a mother, the feeling of motherhood makes you look at life differently, you are responsible for maintaining the marital life and your children’s comfort, you must forget yourself.” (Participant 6)*

### Theme 2: facilitators of disclosure

Few women disclosed perinatal intimate partner violence. Facilitators of disclosure included maternal self-efficacy, threats to security, and formal and informal supportive networks.

### Category 1: maternal self-efficacy

Maternal self-efficacy included high self-esteem and self-empowerment, and having information about violence and individual rights facilitated perinatal intimate partner violence disclosure.

#### High self-esteem and self-empowerment

High maternal self-esteem and empowerment, authority, self-confidence, and financial independence were the factors which facilitate the disclosure of violence for seeking help. One abused woman explained that her high self-esteem facilitated disclosure of violence:

*“I am a woman who can save my life, I can keep my peace, and I can protect my family. I told my condition to the health care provider and I got help from a health center consultant.” (Participant 6)*

#### Having information about violence and individual rights

Most abused women had little information about IPV and, in many cases, did not know they were victims of violence. They considered the husband's violent behavior as normal. Similarly, they had little information on effective strategies to reduce IPV and supportive systems. Maternal adequate information on violence and effective strategies for dealing with violence facilitated violence disclosure. One participant stated that having information about violence facilitated a spouse’s violence disclosure:

*“I know I am a victim of my husband violence. I should not be silent and tolerate his violence….” (FGD- Participant 1)*

Maternal familiarity with individual rights and belief in the necessity of protecting women’s rights, especially in pregnancy, were the effective factors in disclosing intimate partner violence to achieve the rights. One participant described that familiarity with her individual rights facilitated violence disclosure to the forensic organizations and help seeking:

*“Men should know that they have no right to violence against pregnant women. I have come to forensics, I want to make it clear to my husband that he has no right to hit a pregnant woman.” (Participant 9)*

### Category 2: threats to security

The intensity and continuous of violence which threatened women’s physical, psychological and social security facilitated violence disclosure.

#### Intensity of violence

The intensity of violence, such as severe physical, psychological and sexual violence was facilitated violence disclosure. One abused woman explained the severity of violence as an enabler of disclosure:

*“He hit me terribly, he threw me down the stairs, and it was a hard beat. I had severe back pain. I could not endure it anymore, I came to forensic medicine. When I returned home spotting started, and I had an abortion at night.” (Participant 9)*

#### Continuous of violence

Continuous of violence, such as the repetition of violence and frequent physical, psychological and sexual violence facilitated violence disclosure. The continuation of violence which threatened women’s physical, psychological and social security facilitated violence disclosure. One participant explained about the repetition of psychologic violence as an enabler of disclosure:

*“I was always alone. Out of 9 months of my pregnancy, I was alone for 7 months. I got tired. He was always with his friends and did not pay attention to me. I told his mother.” (Participant 4)*

### Category 3: formal and informal supportive networks

Informal su3pportive networks, such as supportive family, supportive friends, and formal supportive networks included establishing effective relationships with the health care system and trust in the judiciary and forensic system could be facilitators of the disclosure of intimate partner violence.

#### Having supportive family and friends

The existence of supportive family and friends for financial support, emotional support, and social support were the effective factors in disclosing violence. One mother described the effect of her family support as a facilitator of violence disclosure:

*“If I talk to my father and give a logical reason that I cannot continue living with my husband, my father will support me so I could break up and take care of my baby with much better quality.” (Participant 11)*

#### Effective social support

Effective social support, including the health care system, the judiciary, and the forensic medicine organization, facilitated the disclosure of intimate partner violence.

Health care system capability to communicate effectively with abused women, their capability to detect perinatal intimate partner violence, their empathetic and empowering approach, the establishment of trust and positive relationship with abused women, and maintain their privacy and confidentially were the effective factors to identify violence and maternal disclosure. One abused woman stated that effective communication of health care and positive relationship facilitated violence disclosure:

*“Every time I went for prenatal care, the midwife asked me if I had a problem with my husband. She was very kind and very supportive, so I told her my hard situation.” (FGD- Participant 5)*

Maternal trust in the judiciary and forensic medicine organization support and maintaining the confidentiality of information in forensic medicine were the effective factors which facilitate intimate partner violence disclosure to seek help. This quote reflects maternal trust in the forensic medicine organization as a violence disclosure facilitator:

*“… My face and ear were injured. My whole body ached. I came for a forensic examination. I do not know what they are doing here, but I am sure they can help me.” (Participant 8)*

## Discussion

This qualitative study investigated barriers and facilitators of perinatal intimate partner violence disclosure. Most of the abused pregnant women concealed perinatal violence, and only a few women disclosed this situation. The results of the study explained several barriers prevented abused pregnant women from disclosing violence.

According to Iranian culture and tradition, the husband is the head of the family, and the wife must obey him. According to the feminist theory and stress related to pregnancy and in terms of present patriarchal social and cultural norms supporting violence, abused women in Iran faced a range of fears such as concern about social judgments, and loss of social reputation that were barriers to disclosing intimate partner violence. Father custody law for children in Iran caused mothers to fear of disclosing violence and its consequences, such as divorce and losing their child. In Iran, divorce is considered the blame for Iranian women. After the divorce, legal custody of the child would be assigned to the father, and one of the most feared consequences of disclosing was maternal fear of losing her child that is aligned with Spangaro et al. (2016) and Garnweidner et al. (2017) studies which emphasized the fear of legal child protection institutions removing their children after disclosure [46, 47]. Some mothers could not protect themselves and their children from the fear of further consequences and of being stigmatized in society because of violence, divorce, and remarriage. Similar to Damra et al. and Mauri et al. studies [48, 49] that highlighted cultural taboo and tradition of protecting the abusive husband as a barrier to disclose violence, evidence from the current study showed that social and cultural norms restrict pregnant and postpartum women from disclosing violence perpetrated by their partners. Other negative consequences of disclosing included the risks of revealing intimate partner violence, fear of retaliation against mothers by husbands, and fear of their own safety. Previous studies have addressed these results that fear of disclosure consequences and maternal safety causes non-disclosure violence in pregnant women [48–50].

Maternal empowerment is a multi-dimensional and dynamic procedure, which enables women to identify their personality and capability regarding all aspects of life. Disclosure is an opportunity for empowering actions that assist abused women in receiving support, care and reduce intimate partner violence [51]. Gashaw et al. (2020) recommended mothers’ empowerment through education, income generating activities, and employment [52]. Through psychological counselling abused women found that they are able to find appropriate solutions to disclose violence and solve intimate partner violence through their empowerment and positive negotiations with their husbands. The result is similar to Dinmohammadi et al. (2021) study that confirmed the effect of self-confidence and empowering women by counseling to reduce the violence and positive talks to their spouses [53]. The maternal familiarity with perinatal intimate partner violence, its danger signs, and the legal aspects of violence, individual rights were identified as facilitators of perinatal partner violence disclosure which is similar to the previous study expressed that it was very important to educate couples and families about violence and its legal aspects [54]. Gebrezgi et al. (2017) showed in their study urgent attention to women’s rights, and health is essential to decrease domestic violence and its risk factors [55].

The women disclosed perinatal intimate partner violence and increased their efforts to protect themselves and their children when they faced with severe spousal violence. Similarly, women disclosed violence when their physical, psychological and social security was threatened. This is consistent with the finding of Sigalla et al. (2018) study suggested that when violence becomes repeated and severe to cause injury, pregnant women decide to disclose it and seek help from the family [56].

The existence of a supportive family for financial, emotional, and social support were the effective factors in disclosing violence which is consistent with the result of previous studies recommended that having social support and family emotional, practical and informational support encouraged pregnant women to overcomes fears of disclosing violence [28, 56].

Positive relationships with the healthcare provider, protection of maternal privacy and confidentiality, and professional and positive verbal and nonverbal behavior of healthcare providers had all been repeatedly identified as facilitators of disclosing partner violence. The result of another study showed women expect health care professionals to ensure their privacy and confidentiality [47]. Health care professionals in Iran were encouraged to routinely ask pregnant women about their experience of spouse’s violence. Intimate partner violence screening made disclosure easier and provided an opportunity for women to talk about violence and being able to get support. Direct questioning motivated most abused women to disclose violence. These results are consistent with previous study which supported direct forms of asking questions [57]. Few women reported that even when directly asked they may decide not to disclose at the initial time of asking, but routine screening provided them with opportunities for disclosure at later prenatal visits, particularly if their husband’s violence intensified during pregnancy or postpartum. The result recommended that pregnant women were more likely to disclose intimate partner violence to health care providers who asked questions in a professional, sensitive and supportive manner and were nonjudgmental, that is aligned with previous studies that supported empathic, nonjudgmental, and professional forms of care which made it easy to talk about violence [49, 57].

Trust in the judiciary and forensic system was another factor that played an important role in the disclosure of intimate partner violence and seeking help. Maintaining the confidentiality of information in forensic medicine was an effective factor to facilitate perinatal intimate partner violence disclosure and seeking help. This result is in contrast to Taherkhani et al. (2017) study, in which unsuccessful help seeking of legal system could be a barrier to disclose violence and led to distrust in legal organizations [28], and Sabri et al. (2015) study that showed negative maternal experiences with the criminal justice system and police [58]. These findings are important because recognizing different aspects of the disclosure will help us develop more comprehensive interventions. The results of this study increase understanding of the barriers to and facilitators of disclosing perinatal intimate partner violence in abused Iranian women. Future studies are recommended to explore Iranian pregnant and postpartum women's coping strategies with intimate partner violence and their needs.

### Strength and limitation

As this study was conducted not only on pregnant and after birth women who choose to disclose intimate partner violence, but also those who did not, the application of this qualitative approach provided unique opportunities, in particular to understand the circumstances which affect the decision to disclose or not to disclose perinatal intimate partner violence. The sensitive nature of the topic in the patriarchal society of Iran was one of the most important limitations of this study. Intimate partner violence was often under-reported in terms of specific socio-cultural norms of Iran and the difficulty to obtain responses from abused pregnant women considering the taboo of violence and its consequences, the stigma associated with reporting violence, and the normalization of violence against women. Similarly, generalizing the findings of this qualitative study to the whole population of abused pregnant women is difficult because of the methodological constraints of the study design.

## Conclusion

Disclosure of perinatal violence is an important and crucial step in the process of finding an effective and sustainable solution for dealing with intimate partner violence and stop the cycle of violence during the perinatal period. If violence is not disclosed, screening and managing of violence may be severely impeded. Recognizing the barriers to and facilitators of violence disclosure play an important role to eliminate the barriers, strengthen facilitators, providing effective supportive services for abused women, and reducing perinatal violence. Focus on the barriers to and the facilitators of the disclosure will be useful to policymakers, health program planners, and health care providers to identify and manage perinatal intimate partner violence, appropriately.

## Supplementary Information

**Additional file 1. **Participants interview guide.

**Additional file 2. **Summary of themes, categories and subcategories emerged from the analysis.

## Data Availability

The datasets used and/or analyzed during the current study are available from the corresponding author on reasonable requests.
