# Supplementary material for: ‘It is a hard decision’: a qualitative study of perinatal intimate partner violence disclosure
Source: Reprod Health. 2022 Nov 14;19:208. doi: 10.1186/s12978-022-01514-7 (PMC9664727; doi:10.1186/s12978-022-01514-7)
Supplement: Supplementary file 1 — Additional file 1. Participants interview guide. [file 12978_2022_1514_MOESM1_ESM.docx]

**Participants interview guide**

Please describe your experience of perinatal intimate partner violence?

What do you feel about intimate partner violence screening during perinatal care?

Could you please talk about the way of your communication with the health care professionals regarding intimate partner violence?

Under which circumstances would you disclose intimate partner violence?

Whom did you tell about intimate partner violence?

What factors help you to disclose intimate partner violence?

What factors prevent you from disclosing intimate partner violence?
