# Supplementary material for: ‘It is a hard decision’: a qualitative study of perinatal intimate partner violence disclosure
Source: Reprod Health. 2022 Nov 14;19:208. doi: 10.1186/s12978-022-01514-7 (PMC9664727; doi:10.1186/s12978-022-01514-7)
Supplement: Supplementary file 2 — Additional file 2. Summary of themes, categories and subcategories emerged from the analysis. [file 12978_2022_1514_MOESM2_ESM.docx]

**Table S1. Summary of themes, categories and Subcategories emerged from the analysis**

| **Subcategory** | **Category** | **Theme** |
| --- | --- | --- |
| Facing with multiple fears | Negative disclosure consequences | Barriers to disclosure |
| Concern about social judgments |  |  |
| Protection of the unborn baby | Protection of family privacy |  |
| Maternal commitments |  |  |
| High self-esteem and self-empowerment | Maternal self-efficacy | Facilitators of disclosure |
| Having information about violence and individual rights |  |  |
| Intensity of violence | Threats to security |  |
| Continuous of violence |  |  |
| Having supportive family and friends | Formal and informal supportive networks |  |
| Effective social support |  |  |
